# A case of insulin and ACTH co-secretion by a neuroendocrine tumour

**DOI:** 10.1530/EDM-13-0082

**Published:** 2014-02-01

**Authors:** S Solomou, R Khan, D Propper, D Berney, M Druce

**Affiliations:** 1 Department of EndocrinologyBarts and the London School of Medicine, QMULW SmithfieldEC1A 7BE, LondonUK; 2 Department of OncologyBarts and the London School of Medicine, QMULW SmithfieldEC1A 7BE, LondonUK; 3 Department of HistopathologyBarts and the London School of Medicine, QMULW SmithfieldEC1A 7BE, LondonUK

## Abstract

A 33-year-old male was diagnosed with a metastatic neuroendocrine carcinoma of uncertain primary. He defaulted from follow-up without therapy and some months later developed episodic severe hypoglycaemia, which was found to be associated with inappropriately elevated insulin and C-peptide levels. It was considered likely that the neuroendocrine tumour was the source of the insulin secretion. Diazoxide and somatostatin analogue were used to control hypoglycaemia. Much later in the course of the disease, he developed metabolic derangement, increased skin pigmentation and psychological disturbance, without frankly Cushingoid physical findings. Investigations revealed highly elevated cortisol levels (the levels having previously been normal) with markedly raised ACTH levels, consistent with the co-secretion of ACTH and insulin by the tumour. Treatment with metyrapone improved his psychological state and electrolyte imbalance. Unfortunately, despite several cycles of first-, second- and third-line chemotherapy from the start of the first hormonal presentation onwards, imaging revealed widespread progressive metastatic disease and the patient eventually passed away. This case highlights the importance of keeping in mind the biochemical heterogeneity of endocrine tumours during their treatment.

**Learning points:**

- The clinical presentation of insulin-secreting tumours includes symptoms of neuroglycopaenia and sympathetic overstimulation.
- Tumour-associated hypoglycaemia can be due to pancreatic insulinomas, and although ectopic hormone production occurs in a number of tumours, ectopic secretion of insulin is rare.
- A possible switch in the type of hormone produced can occur during the growth and progression of neuroendocrine tumours and, when treating neuroendocrine tumours, it is important to keep in mind their biochemical heterogeneity.

## Background

Endocrine tumours may be non-functioning or functioning, with the latter frequently being associated with well-recognised syndromes of specific hormone excess. Insulinomas are neuroendocrine tumours originating from the β-cells of the islets of Langerhans, which produce excess insulin and C-peptide. Insulinomas are rare, with their incidence being estimated to be four per one million person-years (1). Non-islet-cell tumour hypoglycaemia also occurs, most commonly due to glucose utilisation or excess insulin-like growth factor 2 (IGF2) secretion. Tumour-associated hypoglycaemia due to insulin secretion from non-islet-cell tumours is also possible, but very rare. For example, Furrer *et al*. (2) reported a hepatic insulin-secreting tumour.

It is estimated that the ectopic adrenocorticotrophin syndrome (EAS) represents ∼20% of ACTH-dependent and 10% of all types of Cushing's syndrome (3). The more prevalent tumours associated with EAS are bronchial carcinoids, small-cell lung carcinomas, pancreatic tumours, thymic carcinoids, medullary carcinoma of the thyroid and phaeochromocytomas (3). Other tumours contribute to only 5% of all the reported cases of EAS.

Herein, we describe a rare case of both insulin and ACTH secretion by a metastatic neuroendocrine tumour.

## Case presentation

**Figure 1:**
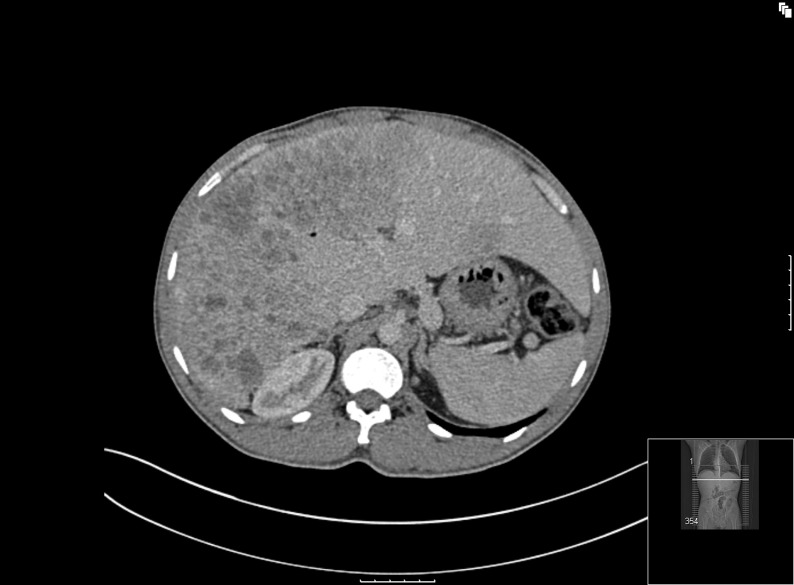
CT scan showing metastatic neuroendocrine tumour involving the liver and gallbladder.

The patient was a 33-year-old male student from Nigeria, with a 10-year history of hepatitis B previously treated with antiviral therapy. He was HIV negative. He became symptomatic in March 2011 with right-upper-quadrant pain. He was otherwise well with no systemic symptoms, in particular, no history of flushing, diarrhoea or wheeze. There was no family history of note, including no family history of endocrinopathy or endocrine malignancy. He had an excellent performance status. On examination, he was found to have hepatomegaly, and an ultrasound scan of the liver confirmed the presence of multiple hyperechoic lesions consistent with malignancy. These findings were confirmed on CT scanning (Fig. 1). Serum markers were negative for a possible hepatocellular carcinoma, and he underwent an ultrasound-guided biopsy. Histology demonstrated a metastatic neuroendocrine tumour involving the liver and gallbladder with Ki67 immunoreactivity of 10% overall but 40% focally. Immunohistochemistry revealed strong positive staining for CAM5.2, CD56, synaptophysin and CDX2, patchy positivity for polyclonal CEA and CD117, and focal staining for CK20 and TTF1. The tissue was negative for PLAP, CD30, HCG, AFP, CK7, PPP and HepPAR. The pattern, in particular, CDX positivity, was felt to favour an intestinal or gastric origin while not excluding a pancreatic primary. The site of the primary tumour could not be determined from the imaging, including detailed review of the pancreatic images. The patient was offered platinum-based chemotherapy, but he initially defaulted from treatment due to academic commitments. He was also commenced on entecavir antiviral therapy.

## Investigation

Six months after his diagnosis, in September 2011, the patient developed episodic confusion, relieved by eating. There were no other systemic symptoms at that time. One such episode was associated with impaired consciousness and resulted in admission to his local hospital. There he had a measured plasma glucose level of 1.6 mmol/l, with a concurrent insulin level of 287 pmol/l and C-peptide level of 2470 U/l, in keeping with inappropriate endogenous insulin secretion. Unfortunately, no sulphonylurea screen was available, but it was noted that he did not have access to any diabetes medications. He was treated symptomatically in the first instance (i.v. dextrose) and also commenced on therapy with diazoxide 100 mg bd. This improved his symptoms to a degree, but he still complained of intermittent confusion associated with low measured capillary blood glucose levels. Radiolabelled octreotide scanning with SPECT was requested, both for staging purposes and for enabling some prediction of therapy response to somatostatin analogues, and he was subsequently prescribed a somatostatin analogue (Sandostatin LAR injections 30 mg every 4 weeks) to control hypoglycaemia, pending the imaging results. The symptoms of hypoglycaemia resolved completely, and blood glucose levels that he was recording no longer indicated any significant hypoglycaemia. Further review of immunohistochemistry from the original tumour sample showed it to be negative for insulin, glucagon, PPP and somatostatin.

**Figure 2:**
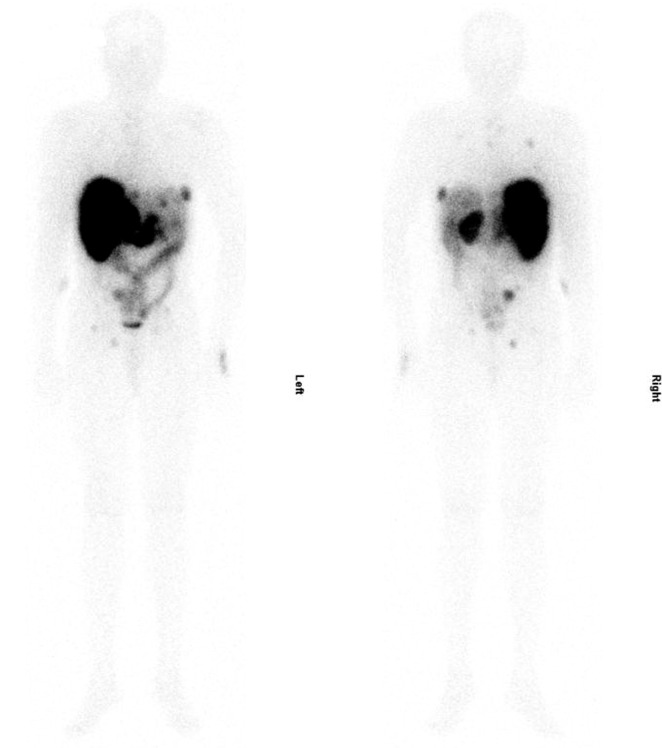
Octreotide scan showing widespread octreotide-avid disease throughout the liver and bony skeleton.

Repeat contrast-enhanced CT scanning revealed radiologically stable disease, and specific scrutiny of the images including the pancreatic views could not confirm the location of the primary. The radiolabelled octreotide scan revealed widespread octreotide-avid disease throughout the liver and bony skeleton (Fig. 2), but no positive sites elsewhere including absent pancreatic uptake. Additional imaging such as pancreatic MRI and more invasive tumour localisation studies such as endoscopic ultrasound were considered, but were not pursued due to the imaging evidence that the tumour was already widespread and of high Ki67 immunoreactivity. Therefore, it was felt that the outcome would not alter the proposed management strategy. At this point, his fasting chromogranin A level was 2061 pmol/l (reference range 0–60). Urinary 5HIAA level was elevated at 684 and 696 nmol/24 h over two consecutive collections. Urinary methoxytyramine level was elevated at 80 000 nmol per 24 h (reference range 0–2500). Urinary metadrenaline and normetadrenaline levels were normal.

## Treatment

At initial re-presentation in September 2011 after the patient defaulted from treatment, CT scans revealed radiologically stable disease, and the patient declined therapy other than somatostatin analogue and diazoxide therapy. With subsequent imaging showing some tumour progression within the liver, the patient agreed to commence chemotherapy in December 2011. Cisplatin and etoposide therapy was commenced on the basis of tumour histopathology. The symptoms of hypoglycaemia remained well controlled and no amendments to the doses of his other medications were required. After two cycles, intolerable side effects required a switch to carboplatin and etoposide therapy, and the patient then completed a further five cycles. His disease remained radiologically stable in the liver and bony sites from the start of chemotherapy until March 2013. He developed an increasing burden of disease in the liver at this point and therefore commenced gemcitabine–carboplatin chemotherapy, receiving five cycles with palliative intention, completing in June 2013. Continued progressive disease with new bony lesions and new extensive peritoneal involvement with deposits in the right and left paracolic gutters led to the institution of temozolomide therapy in August 2013, 140 mg/m^2^ for 5 days orally in 28-day cycles.

In August 2013, he was admitted to the hospital with abdominal pain and fatigue. He had also developed some atypical behaviours and had begun to express some unusual beliefs and some incorrect ideas that the cancer had been cured. On examination, he was found to be cachectic, with darkening of his skin and peripheral oedema. Biochemistry revealed hypokalaemia (1.8 mmol/l), hypomagnesaemia (0.51 mmol/l) and hypocalcaemia (1.73 mmol/l). Vitamin D level was 46 nmol/l (reference range 80–150 nmol/l), and PTH level was 7.5 pmol/l (reference range 1.6–6.9 pmol/l). A CT scan of the brain revealed a small enhancing cortically based lesion (12 mm) in the superior left parietal lobule felt to be consistent with a metastasis, and this was confirmed on MRI. No pituitary abnormalities were found. He had increased skin pigmentation but no clinical features of Cushing's syndrome; however, endocrine investigations revealed a raised plasma cortisol level of >1740 nmol/l (the upper limits of detection of the assay) with a plasma serum ACTH level of 1473 ng/l. He was deemed too unwell to undergo a low-dose dexamethasone suppression test or inferior petrosal sinus sampling. Further review of the immunohistochemistry from the original tumour sample for ACTH staining was not possible due to insufficient tissue being available, and on the basis of the history and biochemical and endocrine findings, a presumptive diagnosis was made of ectopic ACTH secretion from his widespread neuroendocrine malignancy. He was treated with metyrapone and the dose was adjusted following a metyrapone day curve. On a dose of 1 g/1.5 g per 1.5 g taken every 8 h, the levels of cortisol improved with a 0900-h cortisol level of 654 nmol/l and a mean across five samples over the day of 449 nmol/l. This was associated with a vast improvement in his mood and mental state and a full correction of the electrolyte imbalance.

The biochemical findings indicated that the patient's neuroendocrine tumour may have started to co-secrete ACTH along with insulin and that the endocrine perturbations may have been underlying the patient's unusual behaviours. He subsequently also underwent CyberKnife radiotherapy to the brain metastasis.

## Outcome and follow-up

The patient continued being followed by both the endocrinology and oncology departments, but he developed weight loss and peripheral oedema in the context of inexorably progressive malignancy. Unfortunately, he passed away in November 2013. An autopsy was not performed due to his wishes and beliefs and those of his family.

## Discussion

Herein, we describe a patient who presented with a G2/G3 neuroendocrine carcinoma (NEC), with an uncertain primary. Although the majority of extrapulmonary NECs originate in the gastrointestinal tract, in up to 30% of the cases, the primary tumour cannot be located. This patient had a tumour in which some areas were of intermediate proliferation (10% Ki67 immunoreactivity), but with focal areas with proliferation higher than this. The consensus is that such tumours are usually classified by the worst areas; therefore, this tumour would be classified as a G3 NEC. However, consensus guidelines also note that such G3 tumours often have very high Ki67 immunoreactivity and that the patients rarely have hormonal syndromes and usually have negative serum markers (such as CgA) and negative somatostatin-receptor scintigraphy. This was not the case in the patient described herein. It may be that the category contains more than one disease entity for which different therapeutic strategies should be evaluated (4). Therefore, patients with tumours with areas of differing proliferation present a particular challenge in terms of prediction of prognosis and advice regarding treatment. This patient was offered chemotherapy on the basis of the tumour areas of highest proliferative activity, but he defaulted from treatment.

When the patient re-presented, it was due to the development of new symptoms classical of hypoglycaemia and with biochemistry typical of excess insulin production. Tumours secreting insulin are usually of pancreatic origin; however, this was not visualised on cross-sectional imaging or nuclear medicine scanning. Insulinomas are neuroendocrine tumours originating from the β-cells of the Langerhans. It is estimated that only 2% of the insulinoma cases occur ectopically, with the commonest ectopic sites being the duodenum and the immediate vicinity of the pancreas (5). The liver has also been reported as an ectopic site (2). Numerous publications have compared the utility of different investigative modalities to determine the location of small insulinomas to enable accurate surgical targeting; however, such methods are largely redundant in the setting of widespread disease. Pancreatic insulinomas are frequently very small and may not always be visualised by conventional CT and MRI or nuclear medicine techniques. Pharmacotherapy options to control symptoms and for the group of patients with unresectable insulin-secreting tumours include diazoxide (which controls hypoglycaemia by inhibiting insulin release by the pancreas), somatostatin analogues (such as octreotide and lanreotide), and the mTOR inhibitor everolimus (which may act either by causing tumour regression or by having a direct effect on glycaemic control). As somatostatin analogues may also cause worsening of hypoglycaemia, many authorities would trial short-acting agents in the first instance and use longer-acting agents once a benefit had been demonstrated.

The choice of modality for staging is important in these patients. Initial staging in this patient was done by contrast-enhanced CT, which revealed widespread disease. Somatostatin-receptor imaging remains the gold standard for staging neuroendocrine tumours, with the highest sensitivity being observed in head-to-head study of ^111^In-Octreotide imaging (octreoscan), ^131^-MIBG and ^18^FDG-PET. This in turn has an important influence on clinical and treatment decisions. However, for tumours with Ki67 immunoreactivity >15%, FDG-PET imaging has exhibited a higher detection rate for tumours, with 92% sensitivity. It is frequently important to perform ^111^In-Octreotide imaging for such tumours to predict the value of treatment options; however, in circumstances where additional lesions might make a difference to tumour grading or to monitoring of response to therapy, it would seem reasonable to perform FDG-PET. However, more studies are needed to demonstrate whether FDG-PET could be useful for predicting the response to specific therapies (such as chemotherapy regimens), as has been suggested for other cancer types (5).

For a G3 NEC such as that reported herein, particularly presenting with already widespread metastases, current consensus guidelines would suggest chemotherapy, for example, with cisplatin and etoposide (6); however, there are limited data on the clinical history and treatment response in these patients. A very recent retrospective Nordic NEC study has demonstrated a worse response to platinum-based chemotherapy in patients with Ki67 immunoreactivity <55%, although overall they had better survival (7). In a different study, when temozolomide was given to patients as second-line therapy, after platinum-based chemotherapy, patients with Ki67 immunoreactivity <55% exhibited a better response (8).

Ectopic ACTH syndrome (EAS) is also a well-described phenomenon in the setting of neuroendocrine tumours and in fact pro-opiomelanocortin (and ACTH) immunoreactivity can be found in most tissues of the body. Perhaps, it is therefore unsurprising that a variety of benign and malignant tumours of non-pituitary tissue have been found to be associated with EAS (9). Although nearly all neuroendocrine or non-endocrine tumours may be associated with EAS, the more prevalent tumours are bronchial carcinoids, small-cell lung carcinomas, pancreatic carcinoids, thymic carcinoids, medullary carcinomas of the thyroid and phaeochromocytomas (3). When associated with small-cell carcinoma of the lung, or widely metastatic cancer, EAS can develop rapidly with severe features (3). The treatment of choice, once an ectopic source of ACTH is identified, is radical tumour excision if feasible, or the use of inhibitors of cortisol secretion can achieve rapid control of the adverse effects caused by hypercortisolaemia.

Insulin-producing tumours of the pancreas and EAS have been previously reported to co-exist in multiple endocrine neoplasia type 1 (MEN1). For example, Miyagawa *et al*. (10) reported a case of MEN1 consisting of Cushing's disease, primary hyperparathyroidism and insulin glucagonoma. However, in this setting, the two hormones are secreted by two different tumours. The case that we describe herein was not found to be related to MEN1. There was no hypercalcaemia, the CT scans did not indicate the presence of a pituitary mass, the levels of other pituitary hormones were normal, and there was no family history of endocrine disease. Very few cases of insulin and ACTH co-secretion by a single tumour have been described. In 2011, Filippella (11) reported a case of a diabetic patient with a pancreatic endocrine tumour co-secreting insulin and ACTH. Similar to our case, the patient first presented with symptoms related to the presence of an insulin-producing tumour, followed by symptoms related to ectopic secretion of ACTH. Other cases that have been described are from old studies, which do not clearly state whether insulin and ACTH were co-secreted by a single endocrine tumour or by distinct endocrine tumours. An example is the report in 1973 by Sadoff *et al*. (12) which described the case of a patient with functioning islet-cell carcinoma who had amelioration of her hypoglycaemia during the development of the ectopic ACTH syndrome. The co-secretion of these two hormones complicates the diagnosis and management, as they produce a complex clinical picture, where insulin causes hypoglycaemia and EAS exerts opposite effects leading to hyperglycaemia.

Our case highlights the fact that neuroendocrine tumours are pluripotent and a possible switch in the type of hormone produced can occur during the growth and progression of these tumours. The switch in hormone production does not necessarily need to be from insulin to ACTH exclusively. An example is the case reported by Rehman *et al*. (13) where a neuroendocrine tumour presented initially with carcinoid syndrome (producing serotonin) and later as an insulinoma. Moreover, Furrer *et al*. (2) reported a case of a hepatic neuroendocrine tumour, in which the humoral manifestations of the tumour changed during the course of the disease from an extrapituitary acromegaly and a typical carcinoid syndrome to a hyperinsulinaemic hypoglycaemia syndrome. Switches in hormone production by neuroendocrine tumours may be associated with defects in hormonal processing or inefficient secretory mechanisms (13). These switches generally signal tumour de-differentiation and worsening biological behaviour. When treating neuroendocrine tumours, it is important to keep in mind the biochemical heterogeneity of these tumours, which may lead to a mixed clinical picture or even to a change from an indolent tumour to a much more aggressive tumour.

## Patient consent

Written informed consent was obtained from the patient's relatives for publication of the submitted article and accompanying images by their signature of the consent form.

## Author contribution statement

S Solomou is a final year medical student who conducted the literature review and manuscript construction. R Khan is a registrar in endocrinology who provided clinical care for the patient. D Propper is a consultant medical oncologist who provided clinical care for the patient. Prof. D Berney is a consultant pathologist who was responsible for the histology-related aspect of this case and provided clinical care for the patient. M Druce is a consultant endocrinologist who provided clinical care for the patient and was responsible for manuscript construction and advice.
